# Co-presence of human papillomaviruses and Epstein–Barr virus is linked with advanced tumor stage: a tissue microarray study in head and neck cancer patients

**DOI:** 10.1186/s12935-020-01348-y

**Published:** 2020-08-03

**Authors:** Hamda Al-Thawadi, Ishita Gupta, Ayesha Jabeen, Faruk Skenderi, Tahar Aboulkassim, Amber Yasmeen, Mohammed I. Malki, Gerald Batist, Semir Vranic, Ala-Eddin Al Moustafa

**Affiliations:** 1 College of Medicine, QU Health, Qatar University, P. O. Box 2713, Doha, Qatar; 2 Department of Pathology, Clinical Center, University of Sarajevo, 71000 Sarajevo, Bosnia and Herzegovina; 3 Segal Cancer Center/Lady Davis Institute for Medical Research, JGH/McGill University, Montreal, QC Canada; 4 Biomedical Research Centre, Qatar University, P. O. Box 2713, Doha, Qatar

**Keywords:** Epstein–Barr virus, Human papillomaviruses, Oral cancer, Head and neck cancer, Bosnian population

## Abstract

**Background:**

Human papillomaviruses (HPVs) and Epstein–Barr virus (EBV), known oncoviruses, can be co-present and cooperate in the initiation and/or progression of human carcinomas, including head and neck. Based on this fact, we recently reported the prevalence of both HPVs and EBV in cervical and breast cancers.

**Methods:**

We herein explore for the first time the co-prevalence of high-risk HPVs and EBV in 98 head and neck (HN) squamous cell carcinoma (SCC) tissues from Bosnian patients using polymerase chain reaction (PCR) and immunohistochemistry (IHC) analysis, as well as tissue microarray methodology.

**Results:**

The majority of these cancer tissue cases were from the oral cavity (68%). We found that high-risk HPVs and EBV are co-present in 34.7% of the SCC samples; with a significant correlation between the various HPV types and EBV co-incidence (p = 0.03). Our data showed that 30.8% of oral SCCs are positive for E6 oncoprotein of high-risk HPVs and 44.6% are positive for LMP1 of EBV. The most commonly expressed HPVs in our HNSCC samples include HPV types 16, 18, 45 and 58. Additionally, 37.5% of oral SCCs are positive for both HPVs and EBV, with statistically significant association between high-risk HPV types and EBV (p < 0.05). More importantly, our data revealed that the co-presence of HPV and EBV is strongly correlated with advanced tumor stage (p = 0.035).

**Conclusion:**

In this study we show that HPV and EBV oncoviruses are co-present in HNSCC, particularly in oral cancer, where they can cooperate in the initiation and/or progression of this cancer. Thus, further studies are necessary to elucidate the mechanism of this cooperation.

## Introduction

Head and neck (HN) cancers are a group of malignant neoplasms arising from the oral cavity, craniofacial bones, nose, larynx, pharynx as well as the salivary glands [1]. These cancers are the tenth most frequently occurring cancer worldwide [2] comprising of 5–50% of all cancers [3]. The majority of the HN cancers originate from the epithelium lining of the oral cavity, pharynx and larynx, indicating squamous differentiation [1]. Histologically, squamous cell carcinoma (SCC) constitute most tumors found in the head and neck region (~ 90%) [1, 4] followed by other histological types such as lymphomas, sarcomas or blastomas [4]. The main features of this disease include late diagnosis, high mortality rates and morbidity [5, 6].

Tobacco consumption is the primary known cause of HN cancers including oral [5] followed by viral infections by high-risk human papillomaviruses (HPVs) and Epstein-Barr virus (EBV) that are associated with the development and/or progression of head and neck (HN) carcinomas [7–9].

Human papillomaviruses (HPVs) are human oncoviruses that are sexually transmitted and are strongly associated with cervical carcinomas [10]. HPVs are small, double-stranded DNA viruses which tend to infect cutaneous and mucosal epithelial tissues of the ano-genital tract [11]. HPVs are categorized into high-risk or low-risk, with high-risk types duly linked with the onset and progression of cancer [10, 12]. While, low-risk HPV subtypes induce multiplication of epithelial cells that develop into warts or skin papillomas [13, 14]. Earlier investigations indicated that persistent infection with high-risk HPVs is critical for the development of invasive carcinomas [10, 15]. Moreover, it was pointed out that their presence is linked with tumor size, vascular invasion and lymph node metastases [16–20]; which makes them a useful prognostic factor in early-stage cervical, HN, and colorectal carcinomas. In addition, it has been pointed out that E6/E7 oncoproteins of high-risk HPVs convert non-invasive and non-metastatic cancer cells into invasive and metastatic form [21].

On the other hand, Epstein–Barr virus (EBV), a human DNA oncogenic gamma-1 herpesvirus affects around 90% of adults [22]. During EBV infection, cells express six EBV nuclear proteins (EBNA1, -2, -3A, -3B, -3C, and -LP), three latent membrane proteins (LMP1, -2A, and -2B), and multiple non-coding RNAs (EBERs and miRNAs) [23–25]. EBV virus is associated with a broad spectrum of diseases including multiple sclerosis (MS), infectious mononucleosis (glandular fever) and it has the potential to transform B lymphocytes which causes various malignancies, including lymphoid. Additionally, EBV infection is linked with several types of epithelial carcinomas [26]. While, all cases of undifferentiated nasopharyngeal carcinoma are EBV-associated [27], in gastric cancer EBV is present only in a subset of gastric cancers [28, 29]. However, in breast and cervical cancers, the role of EBV is controversial; while a few studies have detected EBV presence in these cancers [30–34], other studies failed to detect it [35–39].

EBV’s key oncogenic protein LMP1 induces cell growth, decreases apoptosis, promotes cell motility and angiogenesis and is also known to express frequently in human oral cancer [40, 41]. Moreover, various EBV genes including latent genes contribute to transformation of human B and some oral epithelial cells and are continually expressed in EBV-associated cancer cells, which links EBV with viral oncogenesis [42–44]; for example, expression of EBNA1, LMP1, -2A genes characterize type II latency and is linked with Hodgkin’s lymphoma and some carcinomas like gastric, nasopharyngeal and breast [22, 26].

Accumulating evidence show that infection with at least one type of high-risk HPV alone is not sufficient to induce neoplastic transformation; high-risk HPV-infected cells must undergo further genetic changes and/or co-infection with another oncovirus to attain complete cellular transformation and consequently tumor development [31, 32, 42, 45–48]. Hence, in this study, we explored the co-presence of high-risk HPVs and EBV in HN cancer samples from Bosnian patients. Our study pointed out that EBV and HPVs are co-present in 34.7% of our samples, and their co-presence is associated with advanced tumor stage.

## Materials and methods

### Sample collection and ethical approval

All samples were taken from formalin-fixed paraffin-embedded (FFPE) tissues from surgically removed and pathologically confirmed squamous cell carcinomas of the head and neck. The samples were retrieved, and approved to be used for research, from the pathology archive of the Department of Pathology, Clinical Center, University of Sarajevo. All samples for the study were de-identified, therefore the patient consent was not needed per local protocol. Only cases having at least one redundant FFPE block were used in the study. The Qatar University Institutional Biosafety Committee (IBC) and Institutional Review Board (IRB) committees approved the project (Numbers: QU-IBC-2018/064 and APP-16/05/2018/GCC). The study was performed in accordance with relevant guidelines and regulations of these committees at Qatar University.

Prior to molecular assays, all hematoxylin and eosin (H&E) slides were re-reviewed by board-certified pathologists (Drs. Semir Vranic and Faruk Skenderi) to confirm the diagnosis and select appropriate samples for tissue microarray and molecular assays (PCR and IHC).

### DNA extraction

DNA extraction from FFPE tissues was performed using Qiagen AllPrep DNA/RNA FFPE Kit (Qiagen, Ltd., Crawley, UK). Briefly, aliquots of 200 μl of samples were digested with 20 μl of K proteinase and 200 μl of AL buffer at 56 °C, for 10 min. DNA precipitation was performed by adding 200 μl of 96% ethanol. DNA was eluted in 200 μl of AE buffer and stored at − 20 °C until further use. The samples collection and DNA extraction were achieved as previously illustrated [49, 50].

### HPV and EBV detection

Twenty-five nanograms of purified genomic DNA from each sample was analyzed for EBV and HPV by polymerase chain reaction (PCR) as previously described [51] using specific primers for LMP1 as well as E6/E7 of HPV types: 16, 18, 31, 33, 35, 45, 51, 52 and 58. MY09/MY11 and GP5 +/GP6 + primers were also used to amplify the L1 region of the viral genome which is commonly used for HPV detection in clinical and histological studies [52]. GAPDH primers (Forward Primer: 5′-GAAGGC-CATGCCAGTGAGCT-3′ and Reverse Primer: 5′-CCGGGAAACTGTGGCGTGAT-3′) were used as an internal control. Primers and analysis was performed as previously described by our group [49, 50].

Briefly, LMP1 gene (Forward Primer: 5′-TTGGAGATTCTCTGGCGACT-3′ and Reverse Primer: 5′-AGTCATCGTGGTGGTGTTCA-3′) was amplified for an initial denaturation at 95 °C for 10 min followed by 40 cycles of 95 °C for 30 s, 61 °C for 1 min, and 72 °C for 1 min. In parallel, HPV gene was amplified for an initial denaturation at 95 °C for 10 min followed by 40 cycles of 95 °C for 30 s, annealing at temperatures ranging from 50 to 62 °C for 1 min depending on each primer’s melting temperature as previously described [49], and 72 °C for 1 min. Samples were finally incubated for 10 min at 72 °C for a final extension. The PCR product from each exon was resolved by using 1.5% agarose gel electrophoresis.

In each experiment, a negative control (sterile water instead of DNA) and a positive control (such as Hela and Siha cell lines) were used.

### Tissue microarray (TMA)

Tissue microarray construction was attained as described previously by our group [31, 32, 53]. Briefly, cancer samples and controls were embedded into a virgin paraffin TMA block using a manual tissue arrayer (Beecher Instruments, Silver Spring, MD, USA). All FFPE samples were de-identified and assembled without any previous knowledge of linked clinical or pathological staging information.

Two TMA cores of 1.0 mm in diameter were sampled from a cohort of 98 FFPE samples from Bosnian patients with confirmed oral squamous cell carcinoma. Later, sections of 4 µm were cut and stained with hematoxylin and eosin on the initial slides to verify the histopathologic diagnosis (cancer tissues). Next, slides of the completed blocks were used for immunohistochemistry (IHC) assays (against E6 and LMP1 of high-risk HPV and EBV, respectively).

### Immunohistochemistry (IHC)

Immunohistochemical analysis investigating the expression of E6 of HPV and LMP1 of EBV were performed using procedures as previously described [53]. To analyze protein expression patterns of E6 and LMP1 in TMA slides, each slide was deparaffinized in graded alcohol, rehydrated and boiled (microwave) in 10 mM citrate sodium citrate solution (pH 6.0) for 10 min. Endogenous peroxidase activity within the rehydrated tissue was blocked with a solution of 3% hydrogen peroxide in methanol for 10 min at room temperature. TMA slides were further incubated for 35 min at 37 °C with primary monoclonal antibodies for E6 of HPV (clones 1–4 and C1P5, Dako Agilent, Carpinteria, CA and Calbiochem, Canada) and LMP1 of EBV using a fully automated immunostainer (Ventana Medical System, Tuscon, AZ). The fully automated Ventana Medical System uses an indirect biotin–avidin system with a universal biotinylated immunoglobulin secondary antibody. The slides were counterstained with hematoxylin prior to mounting. The staining procedures were completed according to the manufacturer’s recommendations. Negative controls were obtained by omitting specific primary antibody for E6 and LMP1 as well as specific blocking peptides from Santa Cruz Biotechnology.

The tumors were considered positive for E6 and LMP1 if cancer cells exhibited positivity ≥ 1% of the cells [32]. In case of LMP1 protein expression (EBV), we also evaluated the presence of viral infection in tumor-infiltrating lymphocytes and stromal cells [32].

### Statistical analysis

Statistical analysis was performed using IBM Statistical Package for the Social Sciences (version 25) and R. Data were calculated as non-parametric files. Spearman Correlation Rank test was used to assess the significance of HPV and EBV association. We utilized χ^2^ test with Yates correction to assess the significance of the association between clinicopathological data (patient’s age, cancer grade and tumor stage) and the co-presence of HPVs and EBV. Statistical significance was achieved at *p *< *0.05*.

## Results

### Clinicopathological characteristics of the cohort

The cohort of head and neck carcinomas included 123 cases and 17 squamous cell carcinomas from other anatomic locations (skin, cervix and esophagus) making a total of 140 cases. After careful assessment, ninety-eight cases had available FFPE blocks for TMA and PCR.

The mean age of patients was 62.8 years (range, 24–91 years), the majority of them were male (72.8%) (Table 1). All cancers in the study are histologically squamous cell carcinomas (SCC). The study included cancers originating from the oral cavity (66.4%) followed by cancer of the larynx (17.9%). Cancers of the pharynx and other anatomic locations (skin, cervix) were rare and comprised only of 3.6% and 10.7% of the cases, respectively (Table 1).

**Table 1:** Clinicopathological characteristics of the cohort of the patients with squamous cell carcinoma (oral cavity and other locations)

Characteristic	Categories	Number (%)
Gender	Male	102 (72.8)
	Female	38 (27.1)
Squamous cell carcinoma histology (by anatomic location)	Oral cavity	93 (66.4)
	Larynx	25 (17.9)
	Pharynx	5 (3.6)
	Other locations (skin, cervix)	15 (10.7)
	Unknown	2 (1.4)
Cancer grade	G1	24 (17.1)
	G2	85 (60.7)
	G3	16 (11.4)
	Unknown	15 (10.7)
Tumor (pT) stage	pT1	41 (29.3)
	pT2	39 (27.9)
	pT3	21 (15.0)
	pT4	30 (21.4)
	Unknown	9 (6.4)
HPV (E6) oncoprotein expression (IHC)	Oral cavity	21 (44.6)
	Larynx	14 (87.5)
	Pharynx	4 (80)
	Other locations (skin, cervix)	1 (16.6)
EBV (LMP1) oncoprotein expression (IHC)	Oral cavity	35 (76)
	Larynx	1 (33.3)
	Pharynx	3 (100)
	Other locations (skin, cervix)	2 (40)
HPV (E6) oncoprotein expression (PCR)	Oral cavity	29 (45.3)
	Larynx	17 (70.8)
	Pharynx	3 (60)
	Other locations (skin, cervix)	3 (60)
EBV (LMP1) oncoprotein expression (PCR)	Oral cavity	50 (78.1)
	Larynx	14 (21.8)
	Pharynx	1 (4.1)
	Other locations (skin, cervix)	3 (60)

**Fig. 1:**
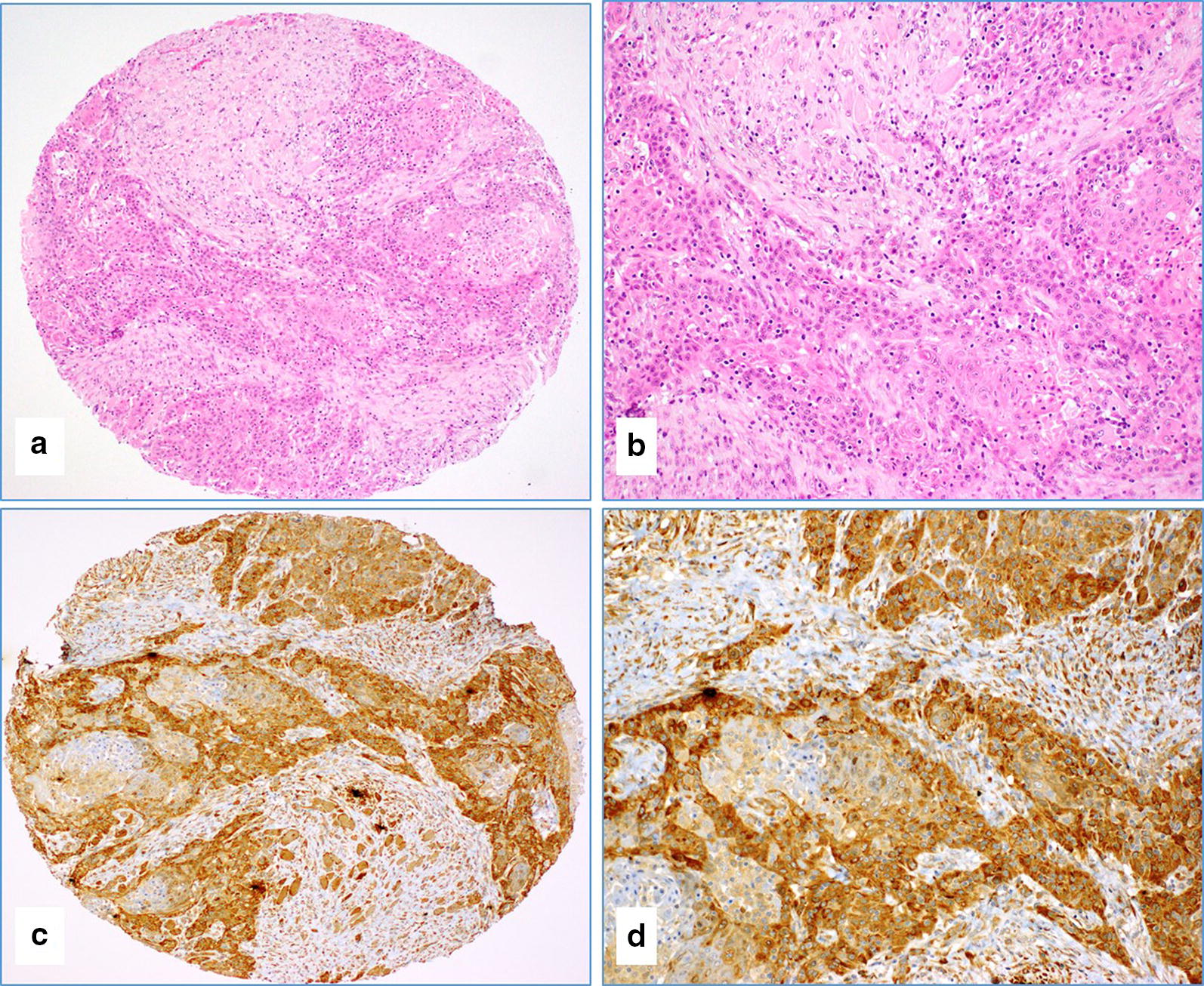
**a**–**d** The case of moderately differentiated (G2) oral squamous cell carcinoma (**a**, **b** Hematoxylin and Eosin staining) with a diffuse and strong expression of E6 protein of HPV by immunohistochemistry (**a** and **c** figures, ×4 magnifications; **b** and **d** figures, ×20 magnifications)

**Fig. 2:**
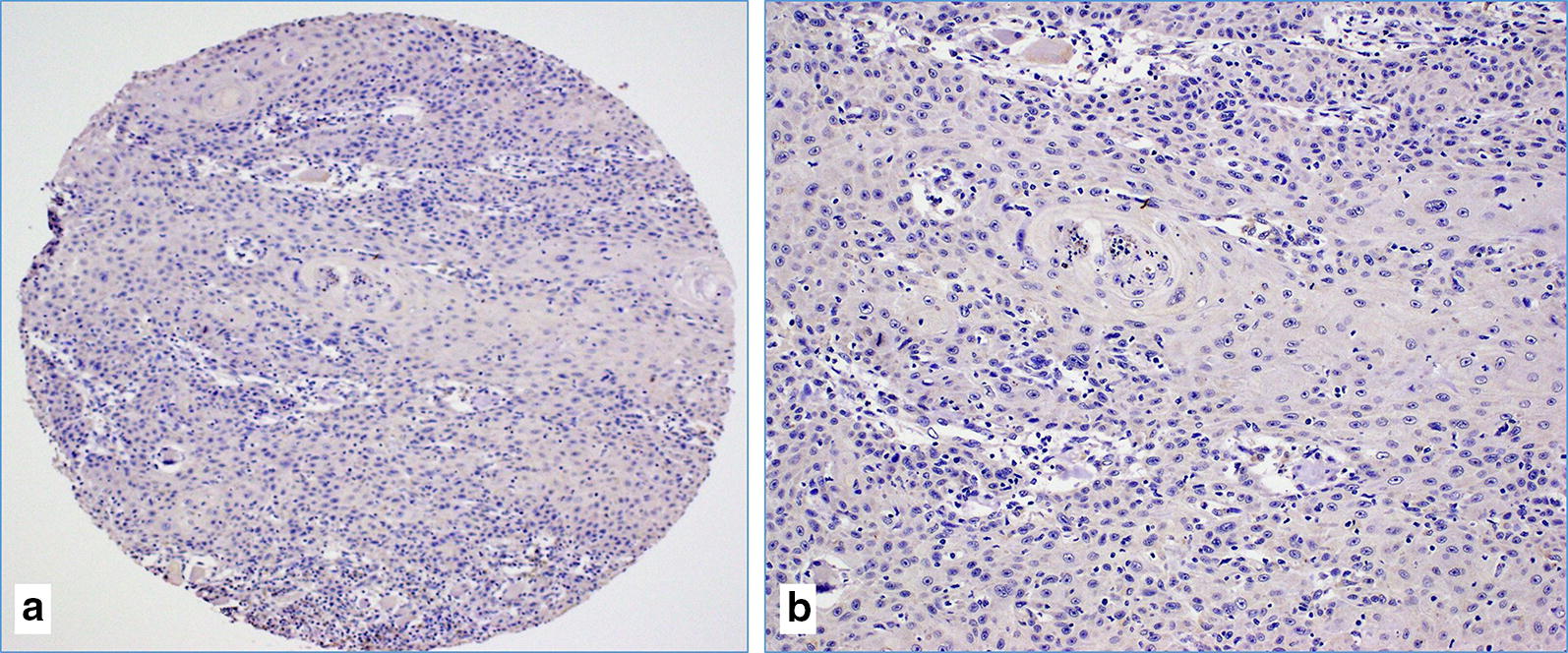
**a**, **b** The case of moderately differentiated (G2) squamous cell carcinoma with negative IHC reaction against E6 of HPV (image A at ×10 magnification and image B at ×20 magnification)

**Fig. 3:**
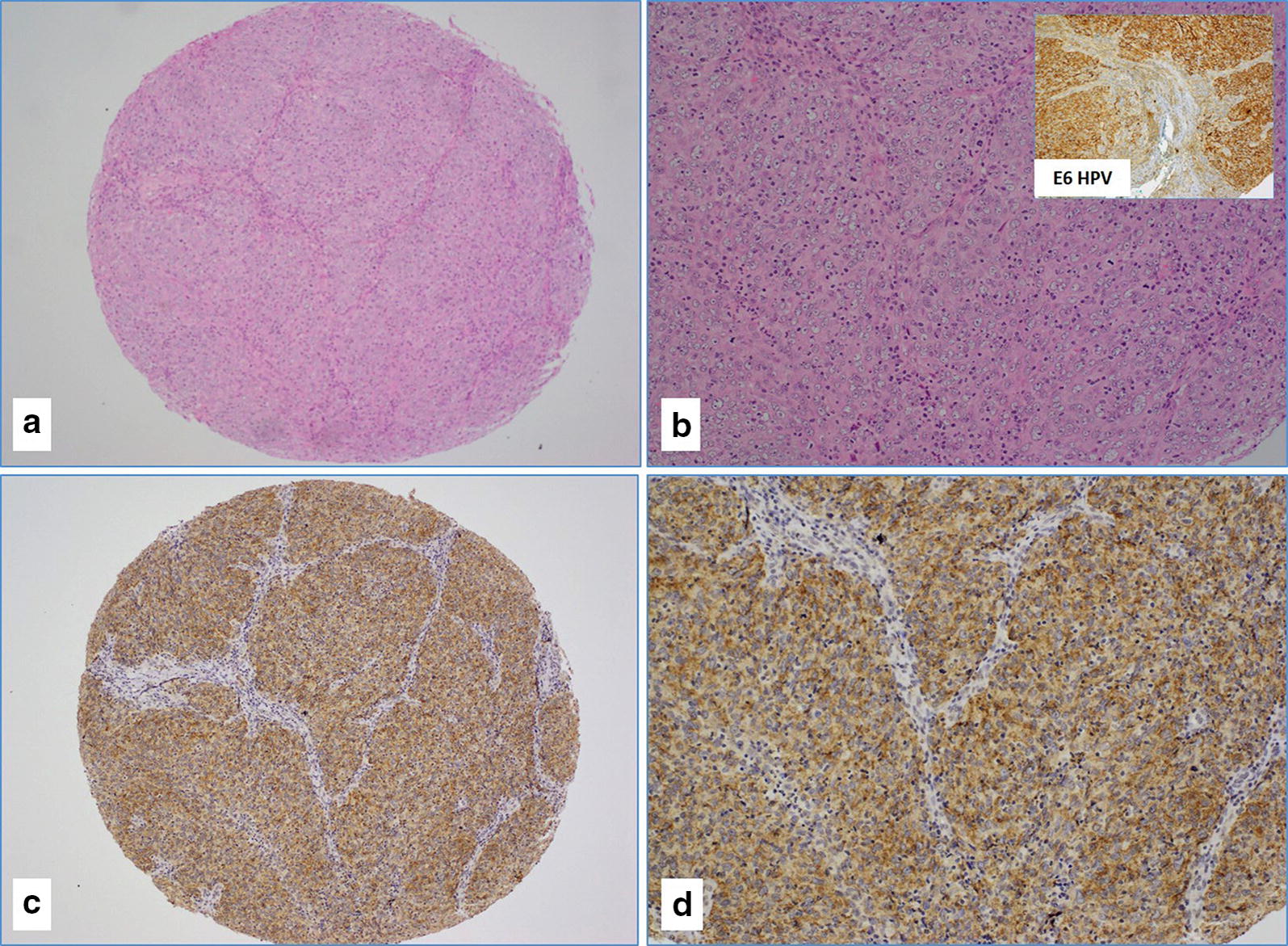
**a**–**d** The case of poorly differentiated (G3) oral squamous cell carcinoma (**a**, **b** Hematoxylin and Eosin staining) that was diffusely and strongly positive for LMP1 protein of EBV (**c**, **d**); This cancer also strongly co-expressed E6 protein of HPV (the image in the right upper corner of **b**) (**a**–**c** ×4 magnifications; **b**–**d** ×20 magnifications)

**Fig. 4:**
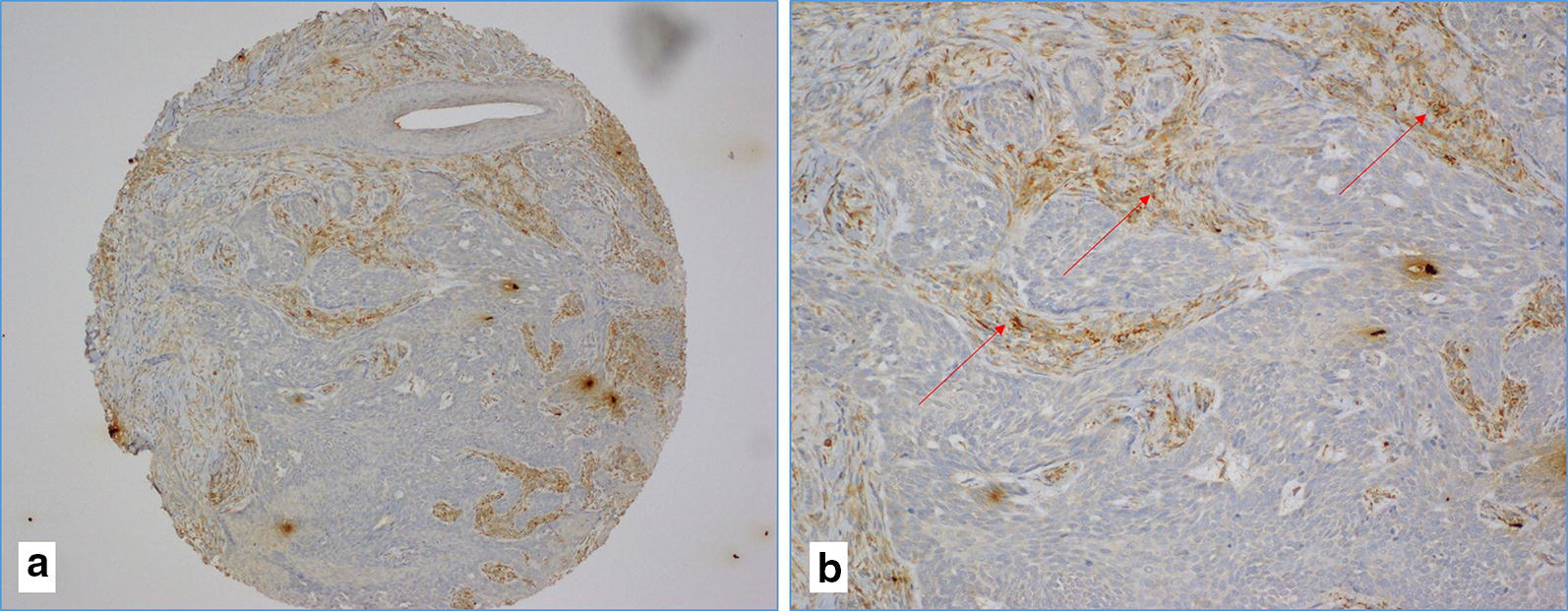
**a**, **b** The case of poorly differentiated (G3) squamous cell carcinoma with negative IHC reaction against LMP1 of EBV (image **a** at ×10 magnification and image **b** at ×20 magnification). The red arrows in image B indicate the positive reaction of LMP1 in tumor-infiltrating lymphocytes

Of the studied cases, there are twenty-four well-differentiated carcinomas cases (17.1%) (G1), 85 moderately differentiated (60.7%) (G2) and 16 poorly differentiated (11.4%) (G3) (Table 1; Figs. 1a, b, 2a, b, 3a, b, 4a, b). Based on the tumor stage, 41 (29.3%) cases are stage 1, 39 (27.9%) cases are stage 2, while, 21 (15%) cases are reported to have stage 3 and 30 (21.4%) cases are stage 4 (Table 1).

### Presence of high-risk HPV subtypes and EBV by PCR

Ninety-eight SCC samples had interpretable results for HPV and EBV by PCR (Additional files 1, 2: Figures S1 and S2).

It revealed that 55 (56.1%) of the samples are positive for E6 of high-risk HPVs and 68 (69.3%) of the samples are positive for LMP1 of EBV. Out of ninety-eight SCC samples, 34 (34.7%) are positive for both HPVs and EBV (Table 2). In addition, there is a significant correlation between the coincidence of EBV with various HPV types [HPV 16 (p = 0.03), HPV18 (p = 0.003), HPV45 (p = 0.04), HPV58 (p = 0.02)] in HNSCC samples (Table 2).

**Table 2:** Prevalence of high-risk HPV types in relation to EBV status in HNSCC cohort (n = 98)

Samples	No. of cases	High-risk HPV types								
		16	18	31	33	35	45	51	52	58
EBV (+)	68	10	31	8	0	0	20	7	10	27
EBV (−)	30	11	24	1	0	3	7	2	4	20
Total	98	21	55	9	0	3	27	9	14	47
*p*-value		0.03*	0.003**	0.34	N/A	N/A	0.04*	0.85	0.89	0.02*

Significant *p*-values are denoted by asterisk (*)

**Fig. 5:**
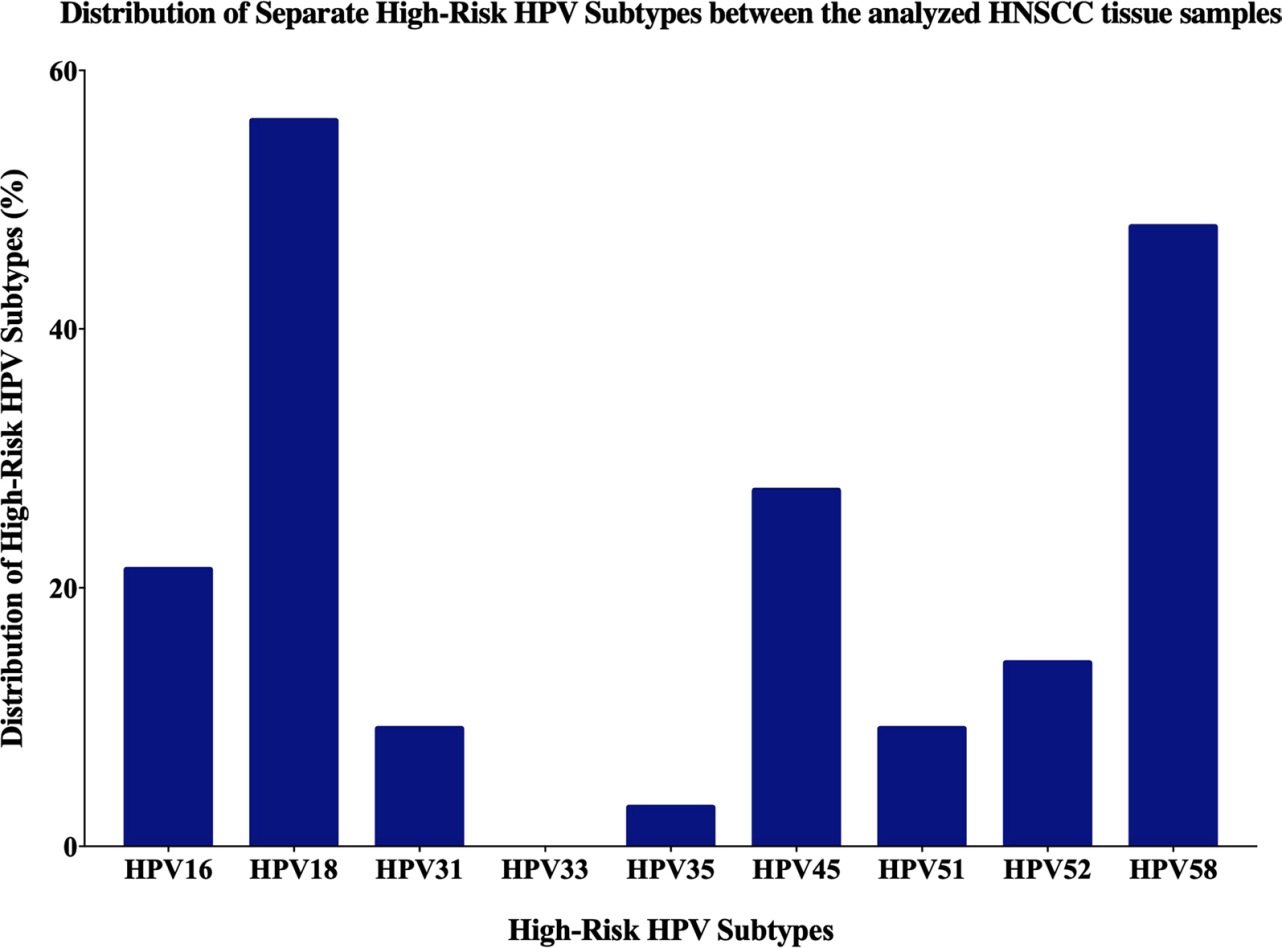
The distribution of each high-risk HPV subtype by PCR according to the frequency in the HNSCC cohort (n = 98)

**Fig. 6:**
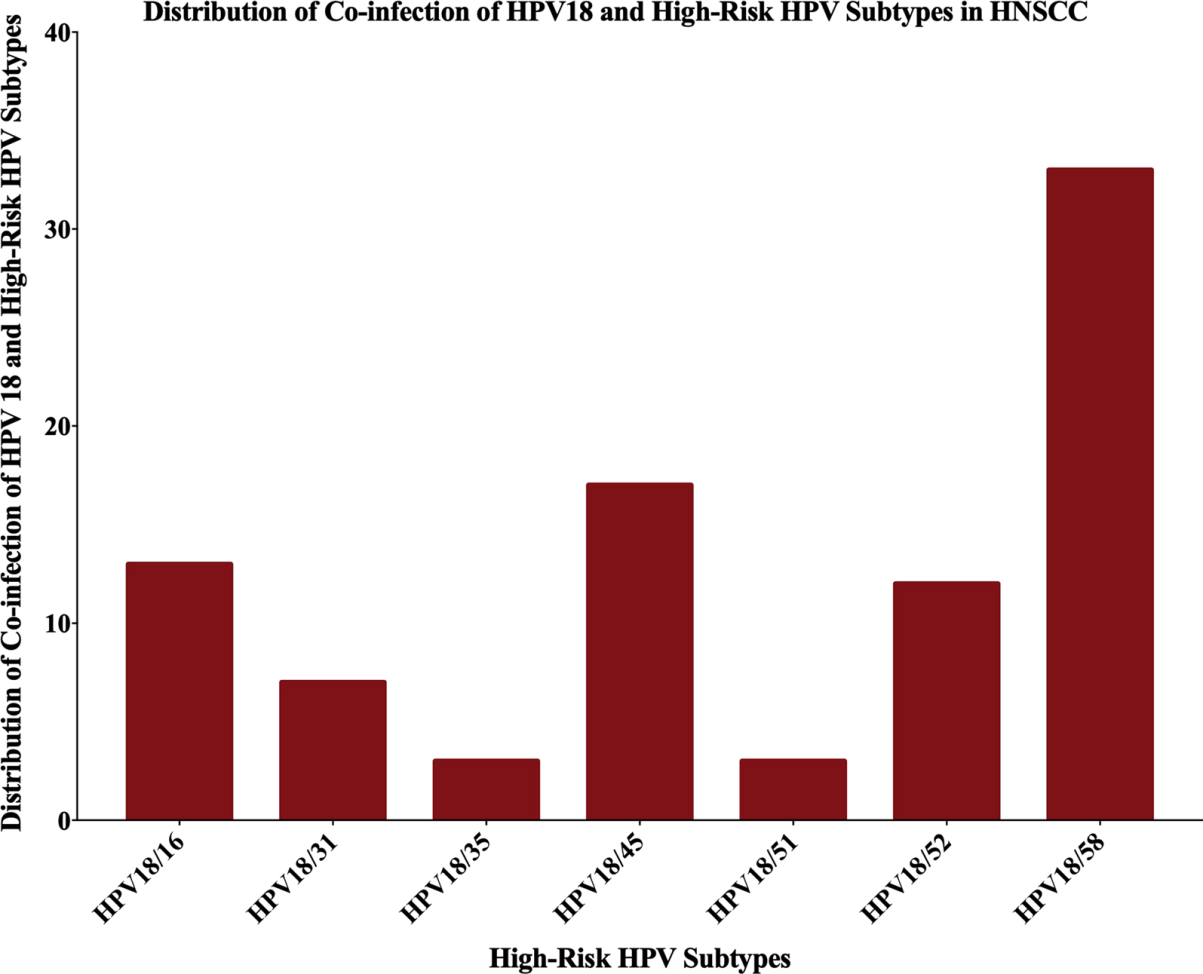
The distribution of co-infection of HPV18 with other high-risk HPV subtypes in HNSCC. The most commonly co-infected subtype was HPV18/58 (33/98; 33.7%) followed by HPV18/45 (17/98; 17.3%), HPV18/16 (13/98; 13.2%), HPV 18/52 (12/98; 12.2%), HPV18/31 (7/98; 7.1%), HPV18/35 and HPV16/51 (3/98; 3.1% each). Since, HPV33 was not present in the cohort, there was no co-expression for HPV18 with these subtypes

Our data showed that, the most commonly expressed high-risk HPVs in Bosnian HNSCC samples are HPV18 (56%) followed by HPV58 (48%), HPV45 (27%), HPV16 (21%), HPV52 (14%), HPV31 and HPV51 (9.2% each) and HPV 35 (3%) (Fig. 5). Moreover, 13 (13.2%) of the 98 samples are co-infected with both HPV16 and 18 (Fig. 6).

Moreover, the Bosnian HNSCC cancers were co-infected with more than one type of HPV in 49 out of 98 samples (50%). Figure 6 shows the most common co-infections of HNSCC samples with HPV18 and other high-risk HPV subtypes.

Regarding the anatomical distribution of these oncoviruses, out of the 98 cases, anatomical location was available for 94 cases. Thus, twenty-nine of the sixty-four oral SCC samples (45.3%) were positive for E6 of high-risk HPVs and 50 (78.1%) of them were positive for LMP1 of EBV. On the other hand, 37.5% of oral SCC samples were positive for both HPVs and EBV. In addition, in oral cancer samples, a significant association was observed between EBV and several high-risk HPV types [HPV-16 (p = 0.000), HPV-18 (p = 0.0005), HPV-31 (p = 0.000), HPV-35 (p = 0), HPV-45 (p = 0.006), HPV-51 (p = 0.000), HPV-58 (p = 0.02)].

### Expression patterns of E6 and LMP1 of high-risk HPVs and EBV

Seventy-nine samples were used for IHC to detect high-risk HPV E6. The remaining 19 cases were not interpretable due to sparsity of cancer tissue in the TMA cores. The frequency of E6 of HPV positivity in these samples by IHC was 57% (45/79), which is in agreement with the PCR data, where 56.1% (55/98) of SCC samples were positive for E6 of HPV (Table 2). Thirteen cases (16.5%) exhibited diffused (90–100%) and strong HPV E6 protein expression in cancer cells (Fig. 1c, d) while ten cases (12.6%) had low HPV E6 expression (1–25% positive cancer cells). The positivity of E6 oncoprotein of high-risk HPV was predominantly observed in cancer cells (Fig. 1c, d). It was both cytoplasmic and nuclear. Rarely, high-risk HPV E6 oncoprotein expression was noted in cancer stroma (fibroblasts and inflammatory cells) (Fig. 1c, d). The remaining 34 cases were negative for E6 of HPV (Fig. 2).

Additionally, using IHC we found that the expression of E6 of high-risk HPV was detected highly in the larynx (87.5%) and pharynx (80%) followed by the oral cavity (44.6%) (Table 1); Results are comparable to PCR data, where HPV was highly expressed in the larynx (70.8%) and pharynx (60%) followed by the oral cavity (45.3%) (Table 1).

LMP1 oncoprotein of EBV was available for 68 cases, as nine cases were not assessable due to sparse or complete absence of cancer tissue. LMP1 was positive in 42 (71%) of the 59 cases which also correlated with EBV PCR data (positivity 69.3%). The mean percentage of LMP1 protein in cancer cells was 46% (range, 0–95%) (Fig. 3c, d). LMP1 was predominantly cytoplasmic and membranous (Fig. 3c, d). No nuclear expression was observed in any of the tested cases. LMP1 protein was occasionally positive in cancer stroma (lymphocytes) (Fig. 4a, b) as well as in immune cells (lymphocytes) of the TMA cores that contained no cancer tissues but normal tissue with some inflammatory cells (data not shown).

LMP1 was also highly expressed in the pharynx (100%) followed by oral cavity (76%) and larynx (33.3%) (Table 1).

Seventeen of the 57 samples (29.8%) were positive (≥ 1% positive cancer cells) for both EBV and HPVs in SCC (Fig. 3b); nevertheless, in our PCR data, co-incidence in SCC samples was 34.7%. Seven cases were both HPV and EBV negative. In addition, we found statistically significant association between various HPV types and EBV co-infection (p = 0.03) in SCC; this data is in concordance with our PCR data.

### Correlation of clinicopathological characteristics with HPV/EBV positivity

HPV positivity correlated strongly with tumor grade (p = 0.02), tumor stage (pT) (p < 0.001) and advanced pN stage (p = 0.045) (Table 3). On the other hand, EBV positivity had no significant association with tumor grade (p = 0.47), tumor stage (p = 0.53) and pN stage (p = 0.14) (Table 3).

**Table 3:** Correlation of clinicopathological characteristics with HPV presence

Degree of differentiation	HPV (E6) positive (%)	HPV (E6) negative (%)	p-value
Tumor grade			
Well-differentiated	6 (8.6)	9 (13)	p = 0.02*
Moderately-differentiated	23 (33.3)	20 (28.9)	
Poorly-differentiated	10 (14.4)	1 (1.4)	
Total	39 (56.5)	30 (43.4)	
Tumor Stage (pT)			
pT1	3 (4.4)	14 (20.8)	p < 0.001*
pT2	10 (14.9)	12 (17.9)	
pT3	13 (19.4)	2 (2.9)	
pT4	10 (14.9)	3 (4.4)	
Total	36 (53.7)	31 (46.2)	
pN Stage			
pN0	11 (29.7)	8 (21.6)	p = 0.045*
pN1	8 (21.6)	2 (5.4)	
pN2	7 (18.9)	1 (2.7)	
Total	26 (70.2)	11 (29.7)	

*Significant *p*-values

More significantly, we found that the co-expression of LMP1 of EBV and E6 of high-risk HPVs (EBV +/HPV +) is associated with advanced tumor stage (p = 0.035) (Table 4); nevertheless, no positive association was found with tumor grade (p = 0.4) (Fig. 3b).

**Table 4:** Correlation of clinicopathological characteristics with EBV/HPV positivity

Stages	HPV ±/EBV ± (%)	HPV +/EBV + (%)	p-value
pT1	16 (29)	2 (3.6)	p = 0.035*
pT2	14 (25.4)	5 (9)	
pT3	4 (7.2)	5 (9)	
pT4	4 (7.2)	5 (9)	
Total	38 (69)	17 (30.9)	

*Significant *p*-values

## Discussion

Oncovirus infections are one of the main underlying sources for the onset and progression of infection-related cancer cases [54, 55]. Viral infections are primarily dominant in developing countries; however, their occurrence cannot be overlooked in developed countries [56, 57].

High-risk HPVs and EBV are important etiological factors in human HN cancer, especially oral; as approximately 35% and 55% of oral cancers are positive for these viruses, respectively [48]. Moreover, several studies have indicated the co-presence of high-risk HPVs and EBV in human oral cancer [42, 48, 58–60] and hence this study aims to investigate the co-presence of high-risk HPVs and EBV in tissue specimens of HN cancer from Bosnia in relation to tumor phenotype. Both EBV and high-risk HPVs share various features in terms of infection of oral epithelial tissue; these viruses infect and lead to epithelial tissue differentiation from the upper aero-digestive tract which stimulates both prolific and lytic phases of HPV’s and EBV’s life cycle, respectively [61].

Approximately 38% of virus-related cancers, display presence of EBV in addition to high-risk HPVs [55]; especially types 16, 18, and 33, which cumulatively infect 80–90% of the population worldwide [48]. Combined oncogenic effects of viral infections can lead to the onset and progression of cancer [48, 55].

In this study, EBV infection was observed in 69.3% of SCC cases by PCR analysis, of which 78.1% was found in the oral cavity, this is in concordance with other investigations in oral carcinomas where EBV infection was found to be higher than 50% [58–60]. Jaloluli et al. [62, 63] examined the presence of EBV in eight different countries and showed an average of 55% EBV prevalence (22% in Yemen to 80% in the UK), suggesting EBV infection in oral cancer to be dependent on geographical region [64, 65].

On the other hand, we found that 45.3% of oral cancer cases are positive for high-risk HPVs, similar to previous studies [60]. The most frequent high-risk HPV types in Bosnian SCC patients are (HPVs-16, -18, -45 and -58) (21.4%, 56.1%, 27.5% and 47.9%, respectively. It is important to highlight that, Iljazović et al. [66] reported that the most frequent HPVs in cervical cancer in Bosnian women are types 16, 18, 45, 33, 51 and 31, respectively. However, there are no studies to date regarding the presence of HPVs in HN cancer, including oral, in Bosnia.

Regarding the co-incidence of HPV and EBV in oral carcinoma, our study showed a higher rate of infection (37.5%) in comparison with other HN carcinomas (larynx and pharynx) including the skin and cervix (< 5%). Furthermore, HPV-18 accounted for the majority of oral carcinomas (45.3%) compared with other HN carcinomas (larynx and pharynx) including the skin and cervix carcinomas (< 3%), which have been previously reported [67]. While, a study by Fregonesi et al. found HPV 16/18 positivity in 40% of Brazilian oral premalignant lesions [68]; Shroyer et al. found 17% of HPV16/18 positivity in samples from Colorado of the USA [69]. Our study showed that 12.5% of our samples are positive for HPV16/18, which is in accordance with a study by Agrawal et al. where they found 10% HPV16/18 positivity in oral epithelial dysplasia in India [70].

As reported earlier, high-risk HPV inhibits changes in the EBV genome in normal oral cells and induces EBV lytic recurrence in differentiating epithelial cells, indicating co-infection with HPV might enhance EBV-directed pathogenesis in the oral cavity [61]. Several studies showed co-infection by high-risk HPVs and EBV in roughly 38% of human nasopharyngeal carcinomas [58, 71, 72]; our study showed comparable results where oral cancer cases were co-infected by high-risk HPVs and EBV in 37.5% of the cases. Moreover, we herein found significant association between the co-presence of EBV and high-risk HPVs in SCC. These results are in agreement with a study performed by Jiang et al. [73] where significant correlation was found between EBV and HPV in oropharyngeal cancers [73]. Although, several investigations have been conducted, nevertheless, the exact role of EBV and HPV interaction in human carcinogenesis has not clearly been identified [74]. While, Makielski et al. [61] suggested that HPV infection in the oral cavity may enhance the capacity of epithelial cells to support EBV life cycle, which can further enhance EBV-mediated pathogenesis in the oral cavity. On the other hand, a study by Guidry and Scott [75] denoted that co-infection by HPV and EBV might escalate EBV persistence either via latency or increased viral replication and by increasing HPV oncogene expression. In this regard, it is well established that high-risk HPV infection alone is not enough to stimulate neoplastic transformation of normal human epithelial cells [45]; thus, HPV could interact with other oncoviruses such as EBV to induce cell transform of different types of human epithelial cells including oral [42, 76], indicating the importance of further genetic changes or co-infection with other oncoviruses. Previous research by our group showed that E6/E7 of HPV type 16 along with ErbB-2, which is upregulated in 30% of oral carcinomas  [77], induce cellular transformation of oral epithelial cells [46] via β-catenin tyrosine phosphorylation by pp60 (c-Src) kinase activation [46, 78]. On the other hand, EBV can infect and immortalize human primary epithelial cells in vitro [45, 79]. Among several known EBV genes, it was reported that LMP1 and BARF1 can induce malignant transformation in rodent fibroblasts  [80, 81], indicating their role as viral oncogenes.

Mellin et al. [82] found a significant association between TNM staging and HPV positivity. Our results are in accordance with this study, by showing that high-risk HPV presence is correlated with poorly differentiated SCC (p = 0.02), tumor stage (p < 0.001) as well as more advanced pN stage (p = 0.045). Moreover, we herein demonstrate for the first time that the co-presence of EBV and high-risk HPVs is associated with advanced tumor stage (p = 0.035); suggesting that high-risk HPVs and EBV oncoproteins can cooperate in the initiation and/or progression of human oral cancer. Therefore, coinfections with high-risk HPV subtypes and EBV can enhance the onset and progression of oral or oropharyngeal tumors [58]. Thus, blocking of EBV lytic replication due to induction of HPV may lead to EBV latency which has been linked with invasive phenotype and deferred differentiation in oral keratinocytes latently infected with EBV [83, 84]. Therefore, HPV-induced EBV latency can be associated with long-term EBV oncogene expression that could contribute in the progression of HPV + oral SCC [85]. In this context, our group, previously pointed out the possible cooperative role of LMP1 and/or EBNA1 genes of EBV as well as E5 and E6/E7 genes of high-risk HPVs in the initiation and/or progression of different types of human carcinomas via the EMT event [47]. In the present study, EBV +/HPV + showed positive correlation with advanced tumor stage (p = 0.035), however, no positive association was found with tumor grade (p = 0.4); thus, lack of association between the co-presence of these oncoviruses and tumor grade can be due to the relatively small sample size.

## Conclusions

In this study, we explored, for the first time, the co-presence of EBV and high-risk HPVs in human HN cancer from Bosnia. Our data revealed that the most frequent high-risk HPVs in HN cancers in the Bosnian population are HPV types 18, 58, 45 and 16, respectively. Moreover, we report that EBV and high-risk HPVs are co-present in 34.7% of our HN cancer samples. More significantly, our investigation pointed out that the co-presence of EBV and high-risk HPVs is strongly associated with advanced tumor stage of HN cancer, which suggests that these oncoviruses can cooperate in the progression of this cancer. Thus, we believe that more investigations with a larger number of samples are necessary to confirm these findings which could be very important in the prevention of HN cancer as well as other EBV and HPVs associated cancers via selecting the right vaccine for specific populations. Meanwhile, mechanistic studies are crucial to elucidate the cooperation role of EBV and high-risk HPVs in the initiation and/or progression of human HN cancer.

## Supplementary information

**Additional file 1: Figure S1.** Representative PCR reactions for HPV-subtypes in 15 different HNSCC patients.

**Additional file 2: Figure S2.** Representative PCR reactions for EBV (LMP1) in 15 different HNSCC patients.

## Data Availability

All data generated or analyzed during this study are included in this published article (and its addiitonal files).

## Abbreviations

DNA: Deoxyribonucleic acid
EBV: Epstein–Barr virus
EBNA: EBV-encoded nuclear antigens
HPV: Human papilloma virus
HNSCC: Head and neck squamous cell carcinoma
IHC: Immunohistochemistry
LMP: Latent membrane protein
PCR: Polymerase chain reaction
TMA: Tissue microarray
