# Supplementary figures and images for: Co-presence of human papillomaviruses and Epstein–Barr virus is linked with advanced tumor stage: a tissue microarray study in head and neck cancer patients
Source: Cancer Cell Int. 2020 Aug 3;20:361. doi: 10.1186/s12935-020-01348-y (PMC7397600; doi:10.1186/s12935-020-01348-y)

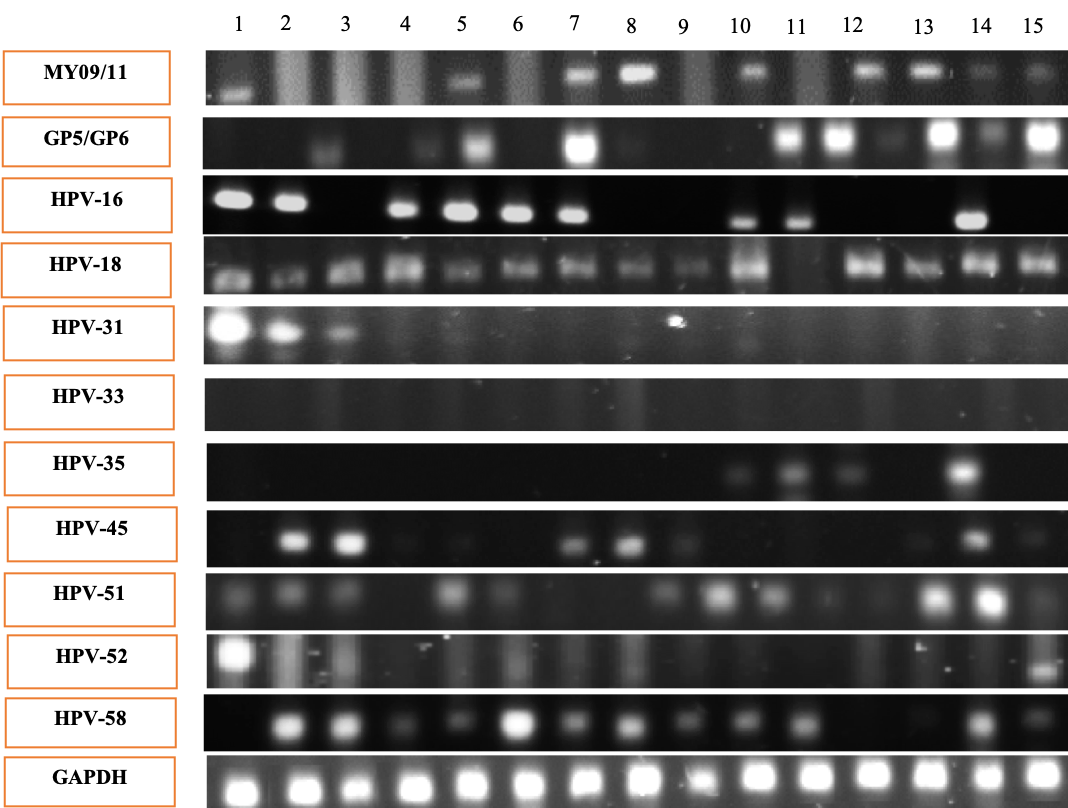

Supplement: Supplementary file 1 — Additional file 1: Figure S1. Representative PCR reactions for HPV-subtypes in 15 different HNSCC patients. [file 12935_2020_1348_MOESM1_ESM.png]

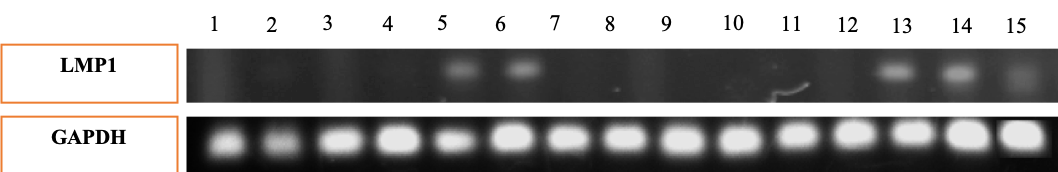

Supplement: Supplementary file 2 — Additional file 2: Figure S2. Representative PCR reactions for EBV (LMP1) in 15 different HNSCC patients. [file 12935_2020_1348_MOESM2_ESM.png]
